# Severe Hydronephrosis and Perinephric Urinoma with Rupture of Renal Fornix Secondary to Postoperative Urinary Retention following Laparoscopic Umbilical Hernia Repair

**DOI:** 10.1155/2016/6754843

**Published:** 2016-07-31

**Authors:** Anthony Dakwar, James Wysock, James Satterfield

**Affiliations:** NewYork Presbyterian-Queens, Weill Cornell Medical College, Flushing, NY 11355, USA

## Abstract

Postoperative urinary retention (POUR) is a known complication following a variety of procedures, with a reported incidence of 2.1–3.8% in general surgery and up to 52% in anorectal surgery. We report a case of POUR in a female resulting in severe unilateral hydronephrosis with a perinephric urinoma due to a ruptured fornix. The extent of hydroureter caused an axial rotation upon itself producing further outflow obstruction. This phenomenon of an anatomical ureter deformity secondary to urinary retention resulting in a ruptured fornix is an unusual occurrence. The patient underwent a percutaneous nephrogram where a stiff guidewire was successfully passed into the bladder by interventional radiology (IR) and allowed for placement of an indwelling ureteral stent. The case presentation, diagnostic evaluation, and therapeutic intervention are discussed.

## 1. Introduction

Postoperative urinary retention (POUR) is a known complication following a variety of procedures, with a reported incidence of 2.1–3.8% in general surgery [1, 2] and up to 52% in anorectal surgery [1, 3]. Most cases of POUR are managed conservatively; however, some will require a one-time catheterization versus long-term catheterization for up to 5 days depending on risk factors [2]. We report a case of POUR in a female resulting in severe unilateral hydronephrosis with perinephric urinoma due to a ruptured fornix. The case presentation, diagnostic evaluation and therapeutic intervention are discussed.

## 2. Case Report 

A forty-three-year-old female with no significant past medical history underwent an uncomplicated, laparoscopic umbilical hernia repair. She had no prior history of lower urinary tract infections, urinary dysfunction, or incontinence. Preoperative computed tomography with intravenous (IV) and per os contrast of the abdomen and pelvis demonstrated normal bilateral kidneys and ureters, with no pelvic abnormalities. Preoperative serum evaluation demonstrated normal renal function with an estimated GFR of 134 mL/min.

A 16 French (Fr) Foley catheter was placed preoperatively without difficulty. Intraoperative details were uncomplicated and the patient received 1200 cc of intravenous fluids. On postoperative day one, her bladder catheter was removed and spontaneous voiding occurred.

On postoperative day two, the patient developed an intestinal ileus and reported inadequate pain control at incision sites that required morphine dosed at 4 mg IV q6 hours. Over the next two days, the patient's ileus had resolved, illustrated by tolerance of regular diet along with passing of flatus and bowel movements. On postoperative day four, the patient complained of dysuria, urinary hesitancy, and right flank pain. Vitals at the time were heart rate of 93 bpm, BP of 138/86 mmHg, respiratory rate of 22, and febrility at 38.2 degrees Celsius. Physical exam elicited a soft but distended abdomen with right costovertebral angle (CVA) tenderness. Significant laboratory values included an increase in white blood cell count to 17,000 and creatinine rise from 0.7 to 5.5 mg/dL. A bladder scan showed a volume of ~600 cc. Thereafter, the patient was catheterized and had a postvoid residual (PVR) of 800 cc.

A renal ultrasound demonstrated moderate right hydroureteronephrosis with a complex perinephric fluid collection. Further evaluation with computed tomography (CT) of abdomen and pelvis without contrast showed severe right-sided hydronephrosis, a perinephric fluid collection, and a dilated ureter down to the level of the bladder. At this time, our differential diagnosis included acute pyelonephritis, obstructive nephrolithiasis, and iatrogenic injury.

**Figure 1:**
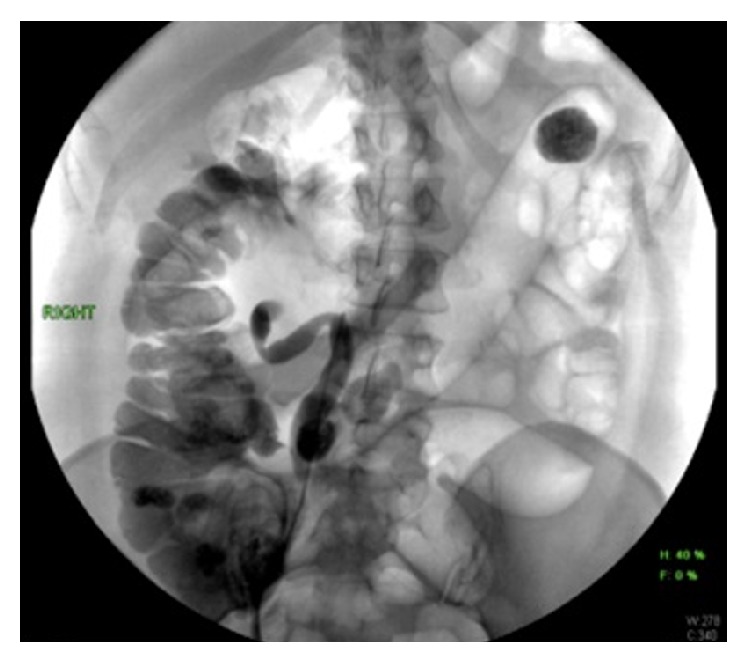
Cystoscopy and retrograde pyelogram demonstrating severe hydroureter.

**Figure 2:**
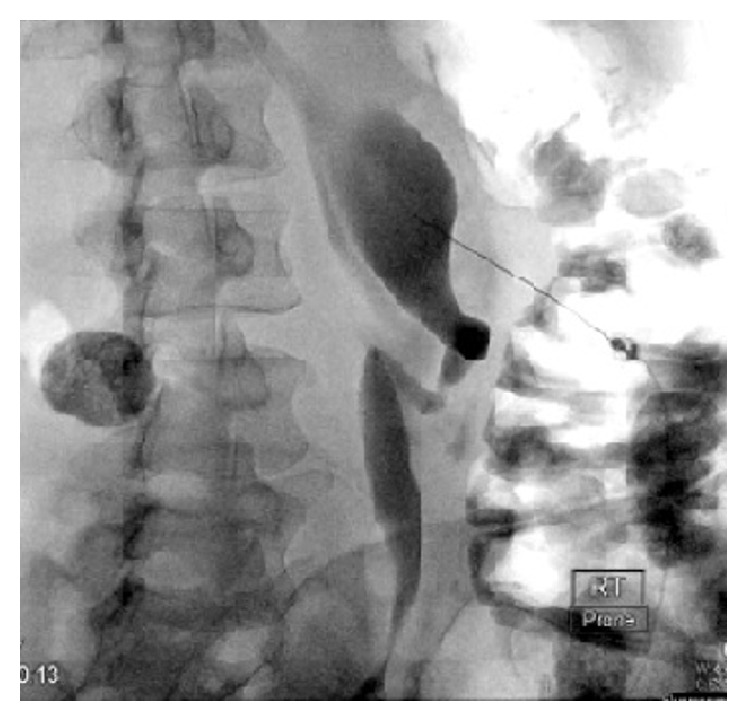
Percutaneous nephrogram illustrating dilated and tortuous ureter.

**Figure 3:**
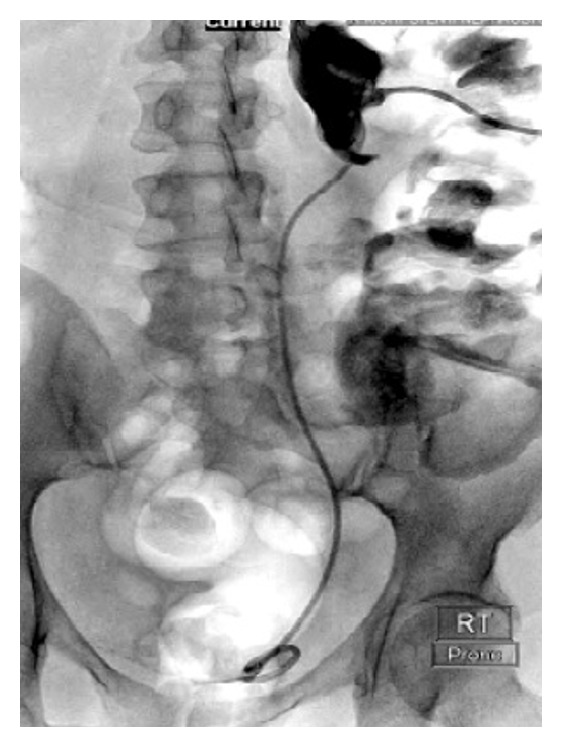
Percutaneous nephrogram illustrating placement of an indwelling 8 Fr double-J ureteral stent.

The patient underwent urgent cystoscopy and retrograde pyelogram that demonstrated severe hydroureteronephrosis and a severely tortuous ureter (Figure 1). Attempts to catheterize the right renal collecting system were unsuccessful secondary to a proximal ureteral tortuosity. The patient then underwent a percutaneous nephrogram where a stiff guidewire was successfully passed into the bladder by interventional radiology (IR) and allowed for placement of an indwelling 8 Fr double-J ureteral stent (Figures 2 and 3). At the end of the procedure, a diversionary 8 Fr nephrostomy catheter was placed into the renal pelvis.

The patient tolerated these procedures without difficulty. On postprocedure day one, the patient's WBC and serum creatinine had normalized. The patient's pain had resolved and her percutaneous nephrostomy tube was capped and removed by IR following stable serum creatinine. The patient was discharged with a bladder catheter and the indwelling stent. The patient has since resumed normal voiding and her ureteral stent has been removed.

## 3. Discussion

The incidence of rupture within the urinary collecting system with concurrent peripelvic extravasation is not common and is usually caused by a ureteral calculus with significant obstruction [4]. The fornix is the most common site of rupture followed by the upper ureter when pressure exceeds a level of 25–75 mmHg [5]. Less common etiology of rupture includes iatrogenic injury, trauma, neoplasm, retroperitoneal fibrosis, and posterior urethral valves in neonates [6–8]. The mechanism involved was described by Hinman as an extreme increase in the degree of pelvic backflow resulting in extravasation of urine by way of the calyceal fornices to the pericalyceal connective tissue [9]. Abdominal and flank pain may be attributed to the chemical peritonitis caused by extravasation of urine in the retroperitoneal space [10], as seen with this patient.

The case described here shows a ruptured right renal fornix secondary to an episode of delayed postoperative urinary retention. The mechanism involved hypothesizes that the grade of urinary retention was significant enough to cause severe unilateral hydroureter that resulted in increased pressure up to the right renal collecting system. The extent of hydroureter caused an axial rotation upon itself producing further outflow obstruction. This phenomenon of an anatomical deformity secondary to urinary retention resulting in a ruptured fornix is an unusual occurrence.

POUR is generally defined as the inability to void in the presence of a full bladder and has been shown to be more prevalent in men than women [11]. Risk factors for POUR have been described repeatedly and include age, gender, and comorbidities such as stroke, diabetes, and neuropathy. In addition, increased intraoperative fluid amounts, pelvic/anorectal surgery, duration of surgery, and perioperative medications such as morphine all have been associated with a higher rate of POUR.

The case described above illustrates a significant morbidity caused by an acute episode of urinary retention. The treatment that was employed was not only therapeutic but also diagnostic in nature. The imaging included in the report illustrates a clear tortuosity of the ureter, which was secondary to the severity of outflow obstruction manifested as urinary retention.

In conclusion, rupture of the urinary collecting system in general is rare. In the event of such occurrences, it is safe to state that CT should be part of the initial steps of diagnosis as it can illustrate defects within the renal pelvis, ureter, and retroperitoneal space. Thereafter, cystoscopy or interventional radiology may need to play a role in the absolute treatment of a ruptured fornix.
